# Burkitt's lymphoma of the colon and bronchi: three case reports

**DOI:** 10.1186/1757-1626-1-15

**Published:** 2008-06-05

**Authors:** Khaled M Musallam, Ali T Taher, Ali I Shamseddine

**Affiliations:** 1 Department of Internal Medicine, Hematology-Oncology Division, P.O. Box 11-0236, American University of Beirut Medical Center, Beirut, Lebanon

## Abstract

**Introduction:**

Burkitt's lymphoma is a form of non-Hodgkin's B-cell lymphoma with more than one identifiable variant. The sporadic form most commonly presents with abdominal lymph node involvement.

**Case reports:**

We herein report on three patients from our experience that presented with either endobronchial or colonic Burkitt's lymphoma. Their clinical presentation mimicked that of other syndromes. After confirmatory pathological diagnosis, the patients had complete remission through the use of an optimal treatment protocol.

**Conclusion:**

Our review of the cases and comparable reports warrants careful workup of such presentations to ensure early diagnosis and therapeutic planning of this highly aggressive disease.

## Introduction

Burkitt's lymphoma is an aggressive form of non-Hodgkin's B-cell lymphoma that is usually diagnosed in children and young adults, and to a lesser extent in middle-aged adults. Endemic, sporadic (non-endemic) and immunodeficient variants have been recognized [1]. Burkitt's lymphomas occurring in non-endemic areas, most commonly in developed countries, are believed to be histologically identical to those in endemic areas, found predominantly in equatorial Africa [2]. They are recognized as small non-cleaved cell lymphomas displaying a starry sky appearance due to the high rate of proliferation and spontaneous cell death. Chromosomal translocations involving the *MYC *oncogene are believed to highlight the hallmark of the disease [3]. However; the clinical presentation of Burkitt's lymphoma will differ depending upon the specific variant. In endemic areas, it usually involves the facial bones, particularly the jaw, maxilla, and orbit, especially in young children [2]. This variant has been associated with Epstein-Barr virus (EBV) infection, as well as frequent concomitant malaria infection [4]. In comparison, the sporadic form tends to present in the lymphoid tissues of the gut, often presenting as masses in the Waldeyer ring or the terminal ileum, or even with massive abdominal involvement. Bone marrow involvement is commonly seen in progressive disease [5]. EBV involvement is reported in around 15–30% of cases [6]. The immunodeficient form is often associated with HIV infection, and may also be seen in post-transplant patients who are chronically immunosuppressed. Generalized lymphadenopathy is usually noted in this variant [7]. We herein report three cases of sporadic Burkitt's lymphoma presenting in a manner different from that commonly described. Comparison with previous similar reports is then discussed.

## Case presentation

### Patient 1

Patient 1 is a 62-year-old woman who presented with a few days history of right facial swelling and dyspnea. The patient underwent left radical mastectomy twenty years prior to presentation for a moderately differentiated stage II_B _adenocarcinoma. Computed tomography (CT) scan of the chest showed a large mediastinal mass 5 × 3.5 cm in dimension invading the superior vena cava, right pulmonary artery, and the right main bronchus, with multiple lung nodularity. Bronchoscopic examination revealed a fungating mass intruding into the orifice of the right main bronchus causing partial obstruction. Pathologic examination of the biopsy sample revealed high-grade, non-Hodgkin, B-cell lymphoma. Immunohistochemical testing was positive for CD45, CD20, CD10, and BCL-6, and negative for CD3, TdT, and BCL-2; which was consistent with Burkitt's lymphoma. Cerebrospinal fluid (CSF) studies were always negative for lymphoma. No evidence of lymphoma was noted in the bone marrow. Chemo-immunotherapy with hyper-CVAD (hyper-fractionated cyclophosphamide, vincristine, doxorubicin, dexamethasone) plus rituximab were given according to the Thomas et al. protocol [8] for 8 cycles. The patient had multiple successive admissions for febrile neutropenia and is currently in complete remission (CR), defined as complete disappearance of all known disease.

### Patient 2

Patient 2 is a 16-year-old boy who presented with signs and symptoms of acute appendicitis. Appendectomy was performed on an emergent basis and the tissue was sent to pathology. Diffuse lymphoreticular infiltration of the mucosa of the appendix penetrating the muscularis of the cecal end and extending to the adjacent fatty tissue was noted. High-grade, non-Hodgkin, B-cell lymphoma was described. Immunohistochemical testing was positive for CD45, CD20, CD10, and BCL-6, and negative for CD3, TdT, and BCL-2; which was consistent with Burkitt's lymphoma. CSF and bone marrow studies were negative. The patient received hyper-CVAD plus rituximab according to the Thomas et al. protocol [8] for 8 cycles. The patient has been in CR after completion of chemotherapy.

### Patient 3

Patient 3 is a 37-year-old male patient who presented with right lower quadrant pain, that is colicky in nature and radiating to the whole abdomen. Rectal bleeding was also reported. CT scan of the abdomen showed a 4.4 × 4.1 cm cecal mass with small adjacent lymph nodes, the largest measuring 1.4 × 0.6 cm. Colonoscopy revealed a large ileocecal mass. Biopsy results showed a diffuse lamina propria lymphoid infiltrate with intermediate size cells that are mildly pleomorphic with prominent central basophilic nucleoli and brisk mitotic activity. Immunohistochemically, high-grade non-Hodgkin B-cell lymphoma that is CD45, CD20, CD10, and BCL-6 positive was noted. The cells were negative for CD3, CD 5, BCL-2. KI-67 proliferation index was positive in all tumor cells. The diagnosis of Burkitt's lymphoma was entertained. CSF and bone marrow studies were negative. The patient received hyper-CVAD plus rituximab according to the Thomas et al. protocol [8] for 8 cycles. Pancytopenia, neutropenia, and thrombocytopenia were the main reasons for three consecutive hospital admissions. Repeat colonoscopy and CT scan 4 months post initiation of chemotherapy showed no evidence of disease.

## Discussion

Sporadic Burkitt's lymphoma accounts for 1%–2% of lymphoma in adults and up to 40% of lymphoma in children in the United States and western Europe [1]. The abdomen, mainly the ileocecal area, is the most common presenting site; the ovaries, kidneys, omentum, Waldeyer's ring, have also been reported. Malignant pleural effusions or ascites have been the presenting signs in some patients. Bilateral involvement of the breasts may occur in association with the onset of puberty or with lactation. Central nervous system (CNS) involvement has also been noted [9]. Lymph node involvement is more common in adults, where as children usually present with extranodal disease [9]. Leukemic Burkitt's lymphoma has also been described and assigned type L3 in acute lymphoblastic leukemias [9]. Bronchial (Table 1) and colonic (Table 2) wall involvement have only been described in few reports including ours.

**Table 1:** Characteristics of endobronchial Burkitt's lymphoma.

Reference	Patient 1 (this report)	Richet-Boe et al. [12]
*Age*	62	52
*Sex*	F	M
*Anatomic location*	Endobronchial	Endobronchial
*Clinical presentation*	SVC syndrome	Dyspnea, cough
*Bone marrow*	Neg	Pos
*CSF*	Neg	Pos
*EBV*	Neg	not done
*Treatment*	Hyper-CVAD + rituximab	CVAD
*Follow up*	CR	Mortality

SVC = superior vena cava; CSF = cerebrospinal fluid; EBV = Epstein-Barr virus; CVAD = cyclophosphamide, vincristine, doxorubicin, dexamethasone; CR = complete remission.

**Table 2:** Characteristics of colonic Burkitt's lymphoma.

Reference	Patient 2 (this report)	Patient 3 (this report)	Dunning et al. [13]	Chang et al. [14]
*Age*	16	37	14	23
*Sex*	M	M	M	F
*Anatomic location*	Appendix	Ileocecum	Ileocecum	Ileocecum
*Clinical presentation*	Acute appendicitis	Abdominal pain, rectal bleeding	Colonic ischemia, cardiac arrest, sepsis	Abdominal pain, Intussusception
*Bone marrow*	Neg	Neg	Neg	Neg
*CSF*	Neg	Neg	Neg	Neg
*EBV*	Neg	Neg	not done	pos
*Treatment*	Appendectomy + Hyper-CVAD + rituximab	Hyper-CVAD + rituximab	Ileocecal resection	Hemicoloectomy + CHOP
*Follow up*	CR	CR	Brain death	CR

CSF = cerebrospinal fluid; EBV = Epstein-Barr virus; CVAD = cyclophosphamide, vincristine, doxorubicin, dexamethasone; CHOP = cyclophosphamide, vincristine, prednisone; CR = complete remission.

The endobronchial involvement in our first patient, with simultaneous mediastinal extension, resulted in what is known as superior vena cava syndrome (SVC syndrome), which is a complication of many mediastinal disease resulting in the obstruction of the superior vena cava with various subsequent signs and symptoms. Malignancy is the etiology in 60% of the cases, with bronchogenic carcinoma having the highest frequency. Small-cell and non-small cell lung cancer account for 22% and 24% of the cases, respectively. Lymphomas involvement is noted in 8% of the cases [10]. Endobronchial involvement of Hodgkin's lymphomas has been previously reported [11]; however, our patient and that of Richet-Boe et al. [12] represent the only Burkitt's cases. Our patient had absence of disease in the bone marrow and CSF and was treated with the addition of rituximab to hyper-CVAD which has been shown to improve outcome in Burkitt's lymphoma, especially in adult patients, with a complete response rate of 86% [8]. Any of these parameters might explain the favorable remission in our patient.

Abdominal symptoms are a frequent initial complaint in Burkitt's lymphoma. However, an acute surgical abdomen is a rare presentation. Our second patient and the patient reported by Dunning et al. [13] presented with signs and symptoms of colonic ischemia resulting from lymphoid infiltration of the bowl wall. The case reported by Chang et al. [14] had an underlying intussusception. The uncommon presentations in the three cases called for emergent surgery. If the diagnosis of Burkitt's lymphoma was initially highly suspected, small tumors that are localized and can be safely resected may benefit from complete surgical resection. However, extensive abdominal involvement with presumed Burkitt's lymphoma or metastatic disease should undergo a limited biopsy followed by chemotherapy rather than an aggressive attempt to resect or debulk the tumor [13]. Mortality after emergency laparotomy in the setting of untreated Burkitt's lymphoma was found to be very high. It was presumed to be due to tumor lysis syndrome which can occur after surgical manipulation of the tumor [15].

## Conclusion

Burkitt's lymphoma is an extremely rapidly growing tumor with a high sensitivity to chemotherapy but in which drug resistance can develop quickly. These dynamic features make prompt diagnosis and initiation of appropriate therapy essential for optimal outcome. Our report and review highlighted some unusual presentations of this tumor that should be kept in mind when compiling a differential diagnosis for similar presentations. Pathologic and immunohistochemical testing remain the only definitive methods of diagnosis. The choice between surgical, chemotherapeutic and chemo-immunotherapeutic treatment should be individualized.

## Consent

Written informed consent was obtained from the patient for publication of this case report. A copy of the written consent is available for review by the Editor-in-Chief of this journal.

## Competing interests

The authors declare that they have no competing interests.

## Authors' contributions

KMM designed the study, compiled the different sections of the report, and helped in writing the manuscript. AIS and ATT drafted the manuscript, revised it critically for important intellectual content and helped in preparing the tables. All authors read and approved the final manuscript.
